# Plant-Based Innovation: Using Kabocha Pumpkin Peels for Sustainable Starch

**DOI:** 10.3390/molecules30163363

**Published:** 2025-08-13

**Authors:** Viviane de Souza Silva, Luna Valentina Angulo Arias, José Ignacio Velasco, Farayde Matta Fakhouri, Rafael Augustus de Oliveira

**Affiliations:** 1 Poly2 Group, Department of Materials Science and Engineering, The School of Industrial, Aerospace and Audiovisual Engineering of Terrassa (ESEIAAT), Universitat Politècnica de Catalunya (UPC BarcelonaTech), 08222 Terrassa, Spain; v152283@dac.unicamp.br (V.d.S.S.); jose.ignacio.velasco@upc.edu (J.I.V.); 2 School of Agricultural Engineering, University of Campinas, Campinas 13083-875, SP, Brazil; lunavale@unicamp.br (L.V.A.A.); augustus@feagri.unicamp.br (R.A.d.O.)

**Keywords:** extraction, waste, characterization

## Abstract

Starch is the main source of carbohydrates in human and animal diets. The extraction of this polysaccharide from unconventional residues of minimally processed foods represents an innovation in the production chain and promotes an appropriate destination for organic waste. Kabocha pumpkin produces minimally processed products, but the discarded peel is not processed and becomes organic waste. In this study, starch was obtained from kabocha pumpkin residues and characterized according to its physicochemical composition, morphology, and thermal properties. Kabocha pumpkin peel starch (KPPS) showed variations in granule morphology. X-ray diffraction analysis revealed about 22% crystallinity. The pasting temperature of KPPS was 69.1 °C and the peak, trough, breakdown, final, and setback viscosities were 5293 cP, 2804 cP, 2849 cP, 3550 cP, and 746 cP, respectively. The stability (120 and 260 °C) observed on the thermogravimetric analysis of KPPS allows it to be used as an interesting ingredient in the production of new foods and non-food products, such as packaging. Moreover, using a product that would otherwise be discarded minimizes residue generation, reducing environmental impact and promoting an alternative source of income for the minimal processing food industry.

## 1. Introduction

Pumpkin is native to South America and belongs to the Cucurbitaceae family, unlike the kabocha pumpkin (Japanese pumpkin or Tetsukabuto), which is a hybrid variety for *Cucurbita maxima* Duch and *Cucurbita moschata* Duch [1]. The global production of pumpkins, squashes and gourds was estimated at 27.96 million tons on an area of about 2.02 million hectares in 2020 [2]. Brazil is a leading country in pumpkin production. The country harvested about 78,671 hectares of these crops, resulting in a total production of 417,839 tons [3]. This segment leads the market in the southern, southeastern, and central-western regions of Brazil. These pumpkins are produced in all regions of Brazil [4].

Pumpkin is increasingly studied due to its notable carotenoid content, which are pigments with antioxidant properties, responsible for the characteristic color of pumpkins and attracting consumers [5]. It has a high potential for industrialization owing to its nutritional value and low production cost, allowing the development of various food products [6]. It is often eaten cooked and sold diced when minimally processed [7]. However, this type of handling generates waste in the fruit and vegetable industry due to the disposal of the peel and/or seeds.

The food industry has sought to process natural products to meet the demand for healthy food, creating visible economic growth and increasing residue generation [8] to meet the needs of a growing population [9,10]. Most of this waste may contain components (proteins, lipids, carbohydrates, vitamins, minerals, and water) that are conducive to microbiological proliferation [11] or for use as a food source for pests and vectors that are responsible for the dissemination of diseases. Additionally, toxic compounds like carbon dioxide, which are harmful to human and animal health, could be produced during the incineration of such waste [8,10,12].

It is understood that food losses are notable, and waste occurs at different stages of the food chain from production through handling, transportation, storage, distribution, and consumption [13]. Therefore, the principles of the circular economy (reduction, reuse, recovery, remanufacturing, and repair) can be applied, as it focuses on the minimization of waste [14]. This suggests that the utilization of vegetable waste (which would otherwise be discarded) for the extraction of starch can be advantageous to the environment, consumers, and the industry by reducing organic waste and promoting the availability of an additional raw material.

Therefore, the reuse of waste, such as peels, contributes to a reduction in environmental impacts and offers opportunities for innovation. Among these opportunities, starch extraction stands out. Starch, a widely used carbohydrate, can be recovered from unconventional sources. This recovery process not only ensures the complete utilization of food sources but also adds value to the resulting waste.

Among carbohydrates, starchy foods are the main source of carbohydrates in the human diet [15], but they can also play an important role in various industries, as shown in Table 1. The characterization of starch is essential to determine its application in industry or at any other direct or indirect consumption. Starch granules are composed of two polymers, amylose (a mostly linear α-(1,4)-linked D-glucose polymer) and amylopectin (a highly branched α-1,4 and α-1,6-linked D-glucose polymer), which together form a semicrystalline granular structure. This is due to the organization of amylopectin into double helices that form crystalline regions, interspersed with amorphous zones where amylose is mainly found. This structure is evidenced by the typical birefringence patterns (‘Maltese cross’) observed under polarized light microscopy, indicating alternating crystalline and amorphous domains [16,17,18]. The crystalline structure is related to the physical state of the starch, which determines its resistance to digestion [19] or processing. This demonstrates the importance of characterizing this macromolecule, as a given type of starch may or may not be modified in the production process.

Obtaining starch from high-quality, unconventional sources of food waste, such as peels, enables the production of this polysaccharide. It also provides an appropriate destination for organic waste and promotes the full use of food for either direct or indirect consumption by the final consumer. However, increased consumption and production of minimally processed products leads to an increase in organic material residues. An alternative to solve this problem is to repurpose these residues, for the development of new products, which reduces the environmental pollution generated by these organic residues. Thus, the objective of this study was to extract and characterize starch from kabocha pumpkin peels, aiming to propose a sustainable and innovative application for this plant-based by-product of minimally processed foods, thereby reducing organic waste and exploring its potential uses in both food and non-food sectors.

## 2. Results and Discussion

### 2.1. Characterization

#### 2.1.1. Chemical Composition

The physicochemical parameters of the starch are given on a dried matter basis in Table 2.

The KPPS sample had a low water content, 3.44%, compared to other starches such as winter squash (15.9%), pumpkin (13.36%) [26], potato (8.52%) [27], corn (6.06%), and rice (8.77%) [28], and below 15% moisture limit for starch according to the Brazilian legislation [29]. The water content of starch influences chemical, biochemical, and physical processes in the food industry, as well as the nutritional value and taste of food products. Due to the low water content in KPPS, the possibility of microbial proliferation in the starch is reduced, making it microbiologically safe for food products.

For the KPPS samples the determined contents were as follows: lipids (0.35%), protein (0.39%), and ash (0.03%). Similar values can be seen in Table 2 for starch isolated from potato (0.22%, lipids; 0.46%, protein; 0.42%, ash) and rice (0.33%, lipids; 0.40%, protein; 0.33%, ash). These values are low enough that they do not to interfere with the starch extraction process. Therefore, they are indicative of the quality of the obtained raw material.

The amylose content of KPPS was about 23.19% (Table 2) and the amount of amylopectin determined by difference was 76.21%. The content of these starch fractions depends on the growing conditions and the plant variety. Compared to other starch sources (Table 2), the amylose/amylopectin ratio in KPPS was higher than that of starches extracted from pumpkin Miben, maize, and rice, but lower than that of potato and winter squash starches. The amylose/amylopectin ratio determines the function of the starch. High levels of amylopectin contribute to the uniformity, stability, and texture of starches, favoring the swelling of granules and promoting stability during food freezing and thawing [30]. These are techno-functional properties of starch in demand by industry (Table 1).

#### 2.1.2. Swelling Power (SP) and Water Solubility Index (WSI)

**Figure 1:**
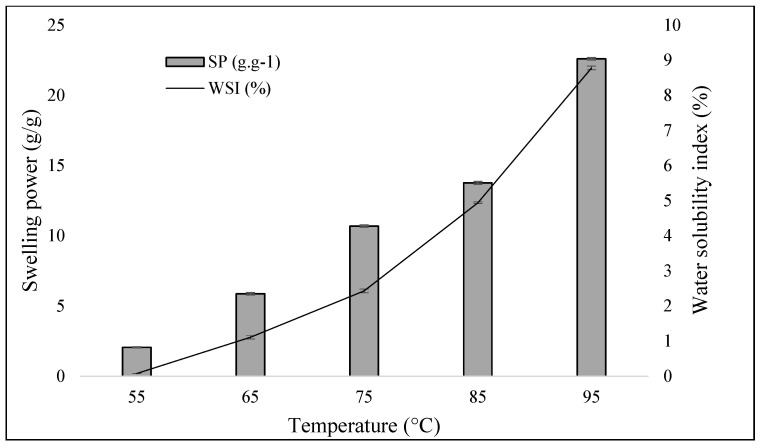
Swelling power (SP) and water solubility index (WSI) of whole kabocha pumpkin peel starch. g/g: weight in grams per gram of water absorbed by the starch. %: percentage of starch solubility in water. Averages described in the Appendix A (Table A1).

The results observed in Figure 1 for the water solubility index and swelling power of KPPS indicate that increasing the temperature from 65 to 75 °C (1.11% to 2.43%, WSI), results in a small increase in swelling power. The water solubility index of KPPS (4.94% at 85 °C and 8.78% at 95 °C (Figure 1)) was lower than that of A-type wheat starch (3.8% at 80 °C and 7.2% at 90 °C) and close to that of B-type wheat starch (7.5% at 80 °C and 13.7% at 90 °C) [31]. However, a steady increase in swelling power was observed as the temperature increased. The value of WSI, however, was close to that of rice (8%) at 95 °C [28]. This difference could be due to the different methods used (e.g., centrifugation conditions and sampling procedures). The effect of temperature on the swelling capacity of starch is induced by the vibration of the molecules at higher temperatures, which breaks the intermolecular bonds and allows hydrogen to form bonds with the water molecule [32]. 

The concentration of amylose in the starch (Table 2) is another factor that influences these properties. Higher amylose concentrations tend to inhibit water absorption by starch granules [31] but, once leached, the absorption capacity improves [33]. Consequently, as the temperature rises, the swelling power also increases, facilitating water penetration into the starch granule and defining the temperature at which this occurs.

### 2.2. Morphology of Starch Granules

**Figure 2:**
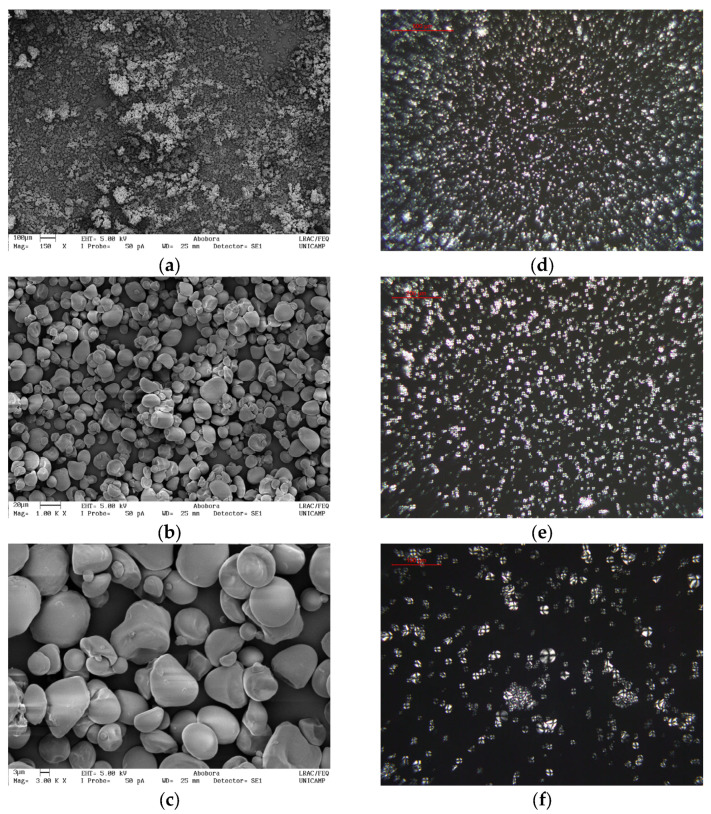
The morphologies of the starch granules under polarized light (PL) microscope and scanning electron microscope (SEM). Scale bar = (**a**) 100 µm, (**b**) 20 µm, and (**c**) 3 µm for SEM photograph and (**d**) 500 µm, (**e**) 200 µm, and (**f**) 100 µm for optical microphotograph.

KPPS exhibited diverse morphological characteristics in its granules, with shapes such as spherical, oval, polygonal, disk, elongated, and kidney-shaped (Figure 2. SEM: a–c). This variation in shape was also described by Liu et al. [34].

There are several variables that affect the morphological characteristics of granules, such as biological origin, cultivation area, harvesting season, water stress, and temperature [35], among other environmental factors [36]. Furthermore, the morphological characteristics of starch granules are associated with the physiological properties of the plant source, including amylose content, swelling power, and water absorption capacity [37,38]. This variation in starch granule form has been described by other authors for other plants, including *Dioscorea* L. species [37]; the corn variety PS_43 and rice variety Kohsar [28]; wheat, potato, and yam [39]; adzuki bean (*Vigna angularis* L.) and edible kudzu (*Pueraria thomsonii* Benth) [38]; the winter squash (*Cucurbita maxima* Duch.) variety Yinli and pumpkin (*Cucurbita moschata* Duch. ex Poir.) variety Miben [26]; and the potato variety Criolla [27].

The variation in starch granule size is likely due to the functional characteristics of the starch and environmental factors during plant development [34,37,38]. Uneven growth within the starch granules or collapse drying may cause differences in granule size [26]. This could also be attributed to variations in the amylose and amylopectin content and their structure, which play a significant role in the size and shape of starch granules [28,38,40]. Matter such as mold, stone, metal, dirt, soil, insects, and fibers were not visualized in the SEM. This is an indication that the production process was effective [41].

Non-gelatinization of starch can be visualized by SEM analysis (Figure 2. SEM: a–c) and confirmed by optical microscopy using polarized light (Figure 2. PL: d–f), as birefringence was detected, displaying the Maltese cross, indicating crystallinity and intact granules. It is noteworthy that birefringence is lost during the gelatinization process [42,43,44]. This finding indicates that the extraction method employed in this study was adequate, as it left the granules intact at a drying temperature of 40 °C, preventing starch gelatinization. This can be confirmed by analyzing the solubility of KHPS starch, in which water absorption by the granules began at 65 °C (>1% of SP). For KPPS, the correlation between temperature and water absorption capacity is positive; that is to say, higher temperature is associated with greater water absorption capacity (Figure 1).

### 2.3. X-Ray Diffraction Analysis

**Figure 3:**
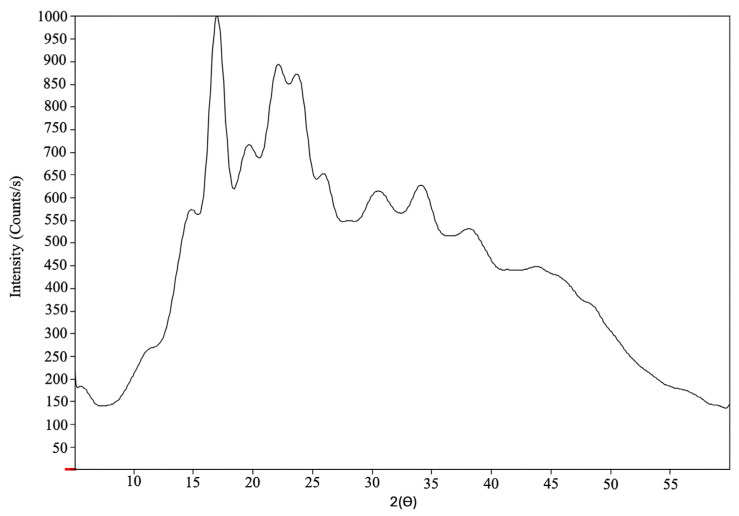
X-ray diffraction pattern of KPPS showing the crystalline fraction (graph peaks) and the total area (amorphous fraction + crystalline fraction) to determine the degree of crystallinity.

Figure 3 shows the X-ray diffraction peaks at 14.96, 17.01, 19.66, 22.15, 23.76, 26.00, 30.57, 34.27, and 37.80°. The X-ray pattern exhibits broad peaks, characteristic of scattering caused by the crystalline material, as explained by Londoño-Restrepo et al. [45] in their X-ray diffraction study of biogenic hydroxyapatite from human, bovine, and porcine bones. The observed pattern indicates that the KPPS structure is hexagonal, consistent with the configuration of type B starch, similar to that reported by Rodriguez-Garcia et al. [46], in which the last one with a hexagonal structure presented among the results obtained some peaks with similar values (14.057, 17.238, 19.623, 22.174, 26.384, 34.334, 37.857°) to those of KPPS. This relationship is supported by experimental data, as evidenced by the viscosity measurements presented in Table 3 and the observation of Maltese crosses in Figure 2, which together indicate characteristics of a crystalline structure.

In addition to identifying the crystalline pattern, the crystallinity index of KPPS was also determined. The crystallinity percentage was approximately 22%, calculated by the ratio of the area under the diffraction peaks to the total diffraction area [36]. This degree of crystallinity is comparable to that found in lotus root (21%), lotus seed (20%), and potato starch (22%) [36], but lower than in rice (34.67%) and corn starch (36.82%) [47]. Such variation in crystallinity levels is commonly attributed to differences in starch composition and botanical origin [48].

These structural differences are relevant because starch crystallinity is often associated with the integrity of the starch granules, which in turn influences their mechanical strength [19]. A lower degree of crystallinity may suggest a higher amylopectin content, as amylopectin contributes to greater viscosity and dough expansion during processing [49]. This relationship is supported by the present study’s results, as evidenced by the viscosity measurements for KPPS shown in Table 3.

### 2.4. Thermal Properties of Starch Granules

**Figure 4:**
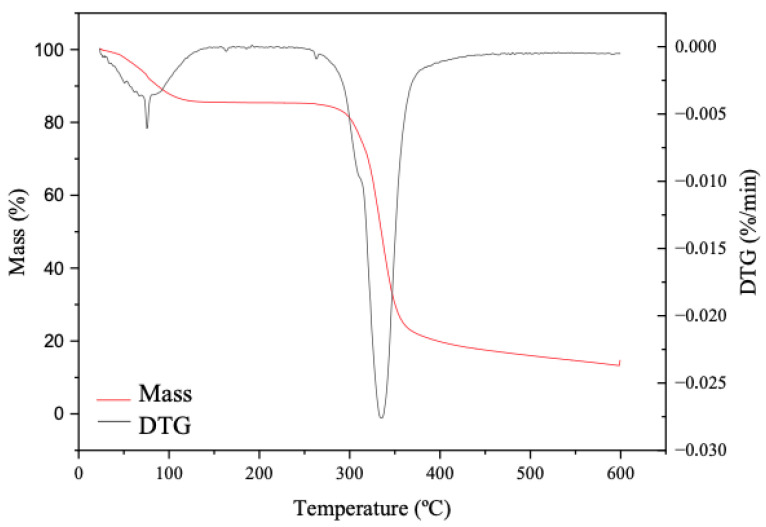
TG/DTG curves for the studied KPPS.

As shown in Figure 4, in general, the material studied was thermally stable in the range of 120 to 260 °C, which occurred with a mass loss variation of about 1%. Below 120 °C, the dehydration of the sample was indicated [50], with a peak around 75.59 °C. This results in a dehydration of approximately 7% by weight, which is similar to what has been observed in other studies [36,51]. The second significant weight loss began about the 260 °C (the beginning of the sample’s degradation) and ended at 360 °C, with a mass loss of about 60% for this temperature range, peaking at around 336.12 °C (Figure 4). Other starches also show maximum weight loss rates above 300 °C, such as *C. auriculatum* (309 °C), potato (325 °C), rice (320 °C), wheat (311 °C), and corn (325 °C) [34].

### 2.5. Pasting Properties

Viscosity is a critical property of starches because, when heated in the presence of water, the granules swell significantly and become more viscous [38]. The stability of the paste is indicated by this change in viscosity during the heating process. Conversely, the changes that occur as the gel cools are indicative of the consistency of the gel and the retrogradation of the starch molecules [52].

KPPS had a lower pasting temperature (69.1 °C) compared to starches from other studies in the literature (Table 3), indicating that it has low resistance to swelling. The peak, trough, breakdown, final, and setback viscosities were 5293 cP, 2804 cP, 2849 cP, 3550 cP, and 746 cP, respectively.

**Table 3:** Pasting properties of kabocha pumpkin peel starch (KPPS).

Parameters			Starches		
	KPPS	* Winter Squash [26]	* Pumpkin [26]	* Edible Kudzu [38]	* High-Amylose Corn [53]
Pasting temperature (°C)	69.1 ± 04	72.28	75.15	86.55	126.6
Peak viscosity (cP)	5293 ± 26	6266	4468	4585	71
Trough viscosity (cP)	2804 ± 19	3876.5	3116	2644	
Breakdown viscosity (cP)	2849 ± 33	2389	1352	1944	49
Final viscosity (cP)	3550 ± 27	4663.5	4075	4372	216
Setback viscosity (cP)	746 ± 42	787.0	959	1736	194

* References of different types of starches for comparison with KPPS (kabocha pumpkin peel starch).

The peak and trough viscosities of KPPS were higher than those of starches from other studies, such as pumpkin and edible kudzu, as well as the peak viscosity for high-amylose corn starch (Table 3). Starches with a high amount of amylose have a higher pasting temperature with a lower peak viscosity. This phenomenon occurs because of the controlled swelling of the starch granules [38]. This can be observed in the case of starch with high-amylose corn (50% amylose) [53] compared to KPPS (23.19%, Table 2).

Compared to winter squash, pumpkin, and edible kudzu starches, KPPS has a lower capacity for retrogradation due to its lower final viscosity. This is because the elevated final viscosity values tend to favor retrogradation after cooling, as they cause leached amylose to recrystallize [26,38,53].

## 3. Materials and Methods

### 3.1. Raw Material

The kabocha pumpkin peel was supplied by Fresh & Freeze Minimally Processed Vegetables industry, located in Sumaré, São Paulo State, Brazil.

### 3.2. Starch Extraction

For the starch extraction, a modified method [54] was applied, which differed in the form of filtration and decanting time of starch. The kabocha pumpkin peel was washed and then mixed with distilled water (1:3) for 2 min using a blender (Magiclean, Arno, São Paulo, Brazil). This mixture was then filtered through a double cheesecloth (325 mesh). Then, the filtrate was transferred to a plastic beaker and refrigerated for 18 h to allow proper the precipitation of starch at the bottom of the beaker. The supernatant was discarded, and the precipitated starch was dried in an oven (Quimis, São Paulo, Brasil) at 40 °C until constant weight. The dried starch was then pulverized using a mortar and pestle, then stored in polyethylene bags until the next step of the experiment.

### 3.3. Characterization

#### 3.3.1. The Chemical Composition of Kabocha Pumpkin Peel Starch

The centesimal composition of water content [55], lipids [55], and ash [56] was determined according to the methods of the Association of Official Analytical Chemists, while protein was determined according to the Kjeldahl method [57] and carbohydrate was determined by difference. All determinations were made in triplicate.

#### 3.3.2. Measurement of Amylose and Amylopectin

The amylose/amylopectin kit (Megazyme, Bray, Ireland) was used to analyze each sample. Amylose content was measured by the concanavalin. The precipitation method used an amylose/amylopectin assay kit (Megazyme), with amylopectin determined by the difference. The manufacturer’s instructions were followed for each step of sample preparation and analysis.

#### 3.3.3. Swelling Power and Water Solubility Index

Swelling power (SP_f_) and water solubility index (WSI_f_) were analyzed using a previous method, with some modifications [58] and adaptations regarding centrifugation. Briefly, the starch sample (W_0_, 0.25 g, db) was weighed into a 15 mL centrifuge tube and suspended in 10 mL of distilled water. The tube was heated at different temperatures (55 °C, 65 °C, 75 °C, 85 °C, and 95 °C), (Dubnoff Bath, SL 157, SOLAB, São Paulo, Brazil) for 30 min with frequent shaking (every 5 min) in a vortex shaker (Fisatom, model 772, São Paulo, Brazil) for 5 sec. The sample was then cooled in an ice water bath before centrifugation (centrifuge Excelsa Baby, model 208 N, São Paulo, Brazil) at 2500 rpm for 40 min. The supernatant was poured into a weighted aluminum pan and dried at 105 °C until constant weight (W_1_). The sediment remaining in the centrifuge tube was also weighed (Ws).

The WSI_f_ and SP_f_ of the flour sample were calculated using Equations (1) and (2):

WSI_f_ = (W_1_/W_0_) × 100% (1)

SP_f_ = (Ws/[W_0_ × (1 − WSI_f_)] (2)

where

SP_f_ = swelling power;

WSI_f_ = water solubility index;

W_1_ = constant weight;

Ws = centrifuge tube weighed;

W_0_ = starch sample initial weight.

### 3.4. Morphology

#### 3.4.1. Scanning Electron Microscopy (SEM)

The morphology of starch granules was visualized using a scanning electron microscope (LEO brand, model Leo 440i, LEO Elektronenmikroskopie GmbH, Oberkochen, Germany) at the Laboratory of Analytical Resources and Calibration (LRAC) of the School of Chemical Engineering—UNICAMP. The powder sample was placed on a double-sided carbon adhesive tape adhered to a metal disk, subjected to the application of a gold layer for thermal conduction, and observed under a scanning electron microscope operated at 5 kV and 50 pA.

#### 3.4.2. Optical Microscopy

Optical microscopy of starch from KPPS was performed using a Leica optical microscope (model: DMLM, Cambridge, UK) at the Laboratory of Analytical Resources and Calibration (LRAC) of the School of Chemical Engineering—UNICAMP. The sample was placed on microscope slides covered with dispersion oil and a coverslip and observed under the microscope under polarized light.

### 3.5. X-Ray Diffraction (XRD)

X-ray diffraction experiments were performed to estimate the crystalline and amorphous fractions of starch from KPPS using an X-ray diffractometer (X’Pert-MPD, Philips, Almelo, The Netherlands) from the Laboratory of Analytical Resources and Calibration (LRAC) of the School of Chemical Engineering—UNICAMP. The starch sample was placed in a circular aluminum cell and exposed to the X-ray beam of the X-ray generator operating at 40 kV and 40 mA, with Cu Kα_1,2_ (λ_1_ = 1.5406 Å, λ_2_ = 1.5444 Å, intensity ratio 0.5) radiation, monochromatic. The 2θ range comprised from 1.0 to 59.9°, with a step size of 0.01° and a fixed time of 1 s per step. The crystallinity index (CI) was estimated according to the method used by Rocha et al. [59], with adaptations, by plotting the diffractogram curve in the X’Pert-MPD data collector software and evaluating the area of the graph using the program OriginPro 8 (Wayne Rasband, National Institutes of Health, Bethesda, MD, USA), where the area above the curve is the crystalline fraction (C_f_) and the area between the curve and the linear baseline is the amorphous fraction (A_f_). The ratio between the areas is the crystallinity index calculated in Equation (3). The graph image was processed and generated in the ^@^Match! program for identification of starch structure.

CI (%) = [C_f_/(C_f_ + A_f_)] × 100 (3)

where,

CI = crystallinity index;

C_f_ = crystalline fraction;

A_f_ = amorphous fraction in the X-ray diffractogram.

### 3.6. Thermal Properties: Thermogravimetric Analysis (TG) and Derivative Thermogravimetry (DTG)

The thermal properties of the starch were measured by thermogravimetric analysis (TGA 50 M Shimadzu, Kyoto, Japan) at the Laboratory of Analytical Resources and Calibration (LRAC) of the School of Chemical Engineering—UNICAMP. The starch sample weight was 10 mg and the heating rate of 10 °C/min. The test was performed from an ambient temperature to 600 °C under N_2_ (30 mL/min) atmosphere [50].

### 3.7. Pasting Properties (Rapid Viscosity Analyzer-RVA)

The viscosity of the starch paste was determined according to method 162 of the International Association for Cereal Science and Technology [60]. The analysis was performed in triplicate, and the results were expressed in centipoise (cP) using an RVA-4500 viscometer (Warriewood, AUS, Sydney, Australia). Curves were plotted and analyzed using TCW3.15.1.255 software. The amount of sample used was 3.0 g.

## 4. Potential Applications

Starch is a decisive ingredient in the formulation of various processed products due to its exceptional technological properties. It acts as a powerful thickening agent, as well as possessing numerous other characteristics [61,62,63], all of which contribute significantly to the textural properties of the product.

In addition, starchy products can also be used in the production of packaging materials, such as films, which can be used not only for food packaging but also as encapsulants for nutrients and/or probiotics [64]. Furthermore, the starch modification technique can be used to obtain properties necessary for application in various industrial sectors. However, to determine the most appropriate starch modification method, it is also necessary to select the intended application.

KPPS has shown resistance to temperatures ranging from 120 to 260 °C (Figure 4), indicating that it can be used in bakery and confectionery products without compromising its properties (Table 4). Its water absorption capacity, solubility, and gel-forming ability indicate that this material could potentially be used in the preparation of sweets, sauces, soups, and ice cream (Table 4).

The concentrations of amylose and amylopectin in the KPPS (Table 2) are another attribute that influences the applicability of this starch, since high concentrations of amylose tend to inhibit the absorption of water by the starch granules; but, after leaching, the absorption capacity is improved [33]. As the temperature of the solution (starch/water) increases, so does the swelling power, which leaches a large portion of the carbohydrates from the starch granule. This process determines both the penetration of water into the starch granule and the temperature at which this occurs (Figure 1). Therefore, the potential application of KPPS in the development of edible packaging or food products could be predicted due to its gel-forming capacity and stability at predetermined temperatures. However, if the properties of the starch in its original form do not meet the desired requirements for application, it can be modified (Table 4) to suit specific industrial applications.

## 5. Conclusions

The main objective of this study was to evaluate the feasibility of extracting starch from KPPS and to characterize the resulting material. The starch granules displayed various shapes, as observed by SEM. TGA analysis showed that KPPS is thermally stable between 120 °C and 260 °C, with a final thermal decomposition temperature of 360 °C. Polarized light optical microscopy detected birefringence and the presence of a Maltese cross, indicating crystallinity and intact granules. X-ray analysis revealed a crystallinity index of approximately 22%, which closely aligns with the amylose quantification result of 23.19%. This analysis (XRD) also identified the starch structure as hexagonal, characteristic of B-type starch. Viscosity analysis showed a paste formation temperature of 69.1 °C. These findings indicate that discarded plant material, such as KPPS, can serve as a valuable source of starch for both food and non-food applications, including for use as a thickener, coating, and biomaterial. By implementing circular economy principles aimed at waste reduction, utilizing kabocha squash peels as a raw material for starch production can create added value and generate income, highlighting several positive implications of this research. While potential applications for this starch have been identified, further studies are needed to verify and confirm its suitability across various uses.

## Figures and Tables

**Table 1:** Application of the starch on the industries.

Industries	Properties Techno-Functional (e.g.)	Reference
Foods	Stabilizers, encapsulation, thickener, texturizers, binder and gelatinization	[17]
Pharmaceutical	Solubilizing, suspending, thickening, preserving, and emulsifying	[16]
Paper	Thickening and gelling agent, colloidal stabilizer, and help form pastes and adhesives.	[20]
Textile	Resilience, elasticity, and resistance.	[21,22]
Packaging	Strength, help form pastes, and stickiness. Physical properties similar to synthetic polymeric material: tasteless, transparent, no odor, and resistance to gases such as O_2_ and CO_2_	[23,24]
3D printing	Improve structural strength, resistance, texture, and gel firmness.	[25]

**Table 2:** Composition of kabocha pumpkin peel starch (KPPS).

Characteristics (%)	Starches					
	* KPPS	Winter Squash [26]	Pumpkin [26]	Potato [27]	Corn [28]	Rice [28]
**Lipid**	0.35 ± 0.01	1.01	1.21	0.22	0.67	0.33
**Protein**	0.39 ± 0.01	0.16	0.89	0.46	0.40	0.40
**Ash**	0.03 ± 0.02	0.19	0.25	0.42	0.20	0.33
**Amylose**	23.19 ± 0.22	30.17	21.35	32.0	7.52	4.90
**Amylopectin**	76.21 ± 0.22	69.83 **	78.65 **	68.0	92.48 **	95.1 **

* Values are expressed as the mean ± standard deviation and are expressed on a dry basis. ** Estimated result based on amylose difference. References of different types of starches for comparison with KPPS (kabocha pumpkin peel starch): winter squash (*Cucurbita maxima* Duch) variety Yinli and pumpkin (*Cucurbita moschata* Duch. ex Poir.) variety Miben [26]; potato variety Criolla [27]; corn variety PS_43; and rice variety Kohsar [28].

**Table 4:** Required characteristics for the use of starch in industry.

Modification	Products	Characteristics	Reference
Reticulation	Bread, pies, samosas, wafers, biscuits, and sausages	Greater resistance to oven cooking temperatures of 120 ≥ 230 °C	[65]
Oxidation	Candy, sweets, and sweetmeat	High clarity or transmittance, Low viscosity Low temperature stability	[65]
Esterification and Crosslinking	Soups, sauces, tomato paste, or ketchup	Clarity of starch paste Greater viscosity Reduced syneresis Freeze–thaw stability	[65]
Hydrolyzed and Esterification	Mayonnaises, salad dressing, ice cream, spreads, and beverages	Lower gelatinization temperature Retrogradation emulsion stabilizers Encapsulation	[62,65]
Pre-gelatinization and Crosslinking	Spaghettis, macaroni, others	Elasticity and softness Delectableness Digestibility. Structural firmness	[62,65]
Pre-gelatinization	Custard, bread mixtures	Increased absorption Retention of water Agglutinant in the meat industry	[62,65]
Grafting	Filmmaking, water-absorbing materials, and textiles	Biodegradability Thermal stability	[62]

## Data Availability

The original contributions presented in this study are included in the article. Further inquiries can be directed to the corresponding author.

## Appendix A

**Table A1:** Swelling power (SP) and water solubility index (WSI) of whole kabocha pumpkin husk starch.

Temperature (°C)	SP (g·g^−1^)	WSI (%)
55	2.06 ± 0.024	0.079 ± 0.001
65	5.87 ± 0.078	1.11 ± 0.049
75	10.69 ± 0.066	2.43 ± 0.055
85	13.77 ± 0.088	4.94 ± 0.026
95	22.6 ± 0.073	8.78 ± 0.054
